# New York State Veterinary Examining Board

**Published:** 1896-04

**Authors:** 


### LEGISLATION.

### NEW YORK STATE VETERINARY EXAMINING BOARD.

At the first examination in September, 1895, only one candidate presented himself, Dr. Comstock, a D.V.S. at Albany, New York. Dr. Comstock passed a brilliant examination and received the first Regents' license. Following are the questions of the second examination held in January, 1896. A number of candidates presented themselves at this examination, but the papers have not yet been made public.

*Anatomy.* Describe the superior maxillary bone of the horse. Describe the frontal bone of the ox. Describe the bones of the hock. Describe a lumbar vertebra. Describe the nuchal ligament. Describe the biceps muscle (coraco-radialis) of the horse. Describe the superficial flexor of the phalanges (perforatus) of the horse. Describe the flexor muscle of the metatarsus. Describe the pharynx of the horse. Describe the stomachs of the ox. Describe the kidneys of the horse. Describe the auriculo-ventricular valves of the heart. Describe the urethra of the horse. Describe the testicles of the bull. Describe the cerebellum of the horse.

*Surgery.* Give the terminations of inflammation. Give the symptoms of obstruction of the duct of Stenson. Name the tissues cut in bleeding from the jugular vein at the upper third of the neck, middle third, and lower third. Describe the operation of puncture of the cæcum. Give the symptoms and pathology of strain of the flexor pedis tendons. Give the pathology and prognosis of ring-bone. Give the structures involved in fistula of the withers. Give the pathology of thrush. Describe the operation of docking (amputation of tail). Give the treatment of articular synovitis of fetlock (anterior wind-galls). Give the treatment of spavin. Give the diagnostic symptoms of navicular disease. Give the treatment of luxation of the patella. What hobbles should be used and what precautions should be taken to prevent fracture of the back? Describe the process of the healing of wounds (cicatrization).

*Physiology and Hygiene.* Give the two classes of organic food substances and state the principal use of each. Name *five* principal substances found in blood. State fully the functions of the blood. Give the normal pulse of the horse, ox, sheep, dog, cat, respectively. What are the differential features of digestion in the horse and in the ox? What are the principal inorganic substances used as food? Explain the use of *two* of these substances. What is *glycogen* and what organ elaborates it? Give the process of digestion in the stomach and in the intestines. Give in brief the physiology of the secretion of urine; of saliva. State the specific gravity of each. Describe the circulation of the blood. Explain *three* ways in which waste matter is excreted from the system. Define *voluntary muscle*, *involuntary*

*muscle, the phenomenon of irritability.* State briefly the functions of the spinal cord. Name the centres in the medulla. State the functions of the fourth, fifth, tenth, and twelfth cranial nerves, respectively.

*Chemistry.* What acid contains chlorin as an important element? What is *organic chemistry?* What is the composition of the atmosphere? Of what are the common forms of urinary calculi (in the horse) composed? What is *albumin?* Mention a substance containing albumin (*a*) as a liquid, (*b*) as a solid. What is an *alkaloid?* Mention *three* well-known alkaloids. What is *glycerin?* Give its chemical properties. How is it obtained? Describe a test for arsenic. What chemical change takes place in the air when inhaled? Describe a test for sugar in the urine. Mention the antidotes that should be prescribed in cases of poisoning from (*a*) caustic alkalies, (*b*) carbolic acid, (*c*) opium. Describe a test for albumin in the urine. What is *oxygen?* How is it obtained? Give the formula and the name of the most common salt of sodium. What is *boracic acid?* How is it obtained.

*Obstetrics.* What are the four chief functions of the generative system of mammals? Name some of the causes of sterility in the mare and in the cow. Describe the placenta of the cow, mare, bitch. Give the symptoms and treatment of persistence of urachus. What is persistence of the urachus? Is it more frequent in solipeds than in ruminants? Give the symptoms and treatment of paraplegia in the cow. Give the symptoms and course of parturition in the mare and in the cow. Give the symptoms, causes, and treatment of retained placenta. Give the symptoms and treatment of post partum hemorrhage. Describe metrotomy in the mare. Give the symptoms, causes, and treatment of leucorrhœa in the mare and in the cow. Describe the pelvis of the mare. Give the causes, symptoms, and treatment of umbilical hernia. Give the treatment of hock presentation. Give the symptoms and treatment of œdema of the umbilicus.

*Pathology, Diagnosis, and Practice.* Give the causes, symptoms, and treatment of hæmoglobinuria in the horse. State the pathology of leucocythæmia. What are its symptoms? State the relative susceptibility to suppuration of the different genera of domestic animals. What are the symptoms of verminous colic in the horse? Describe the means of prevention and method of treatment. What animals suffer from thrush in the mouth? State the cause of this and give the treatment for it. Give the causes, symptoms, and treatment of acute rheumatism in the horse, in the ox, and in the dog. Under what conditions are gall-stones formed in domestic animals? Give the symptoms and treatment. Describe the appreciable changes in capillaries, tissue nuclei, and tissues in inflammation. What is an inflammatory *new formation?* What determines the kind of tissue formed? State the effect of a physiologic dose of mallein on a glandered horse. Describe the mode of using it. Name the usual seat of impaction of the intestine in the horse and state its diagnostic symptoms. Give a rational treatment. Give the life-history of the tænia medio-canellata, with symptoms, treatment, and prevention. What animals harbor the tænia fimbriata? Give the symptoms and treatment. Give the symptoms of hog cholera, the means of propagation and of prevention. State the diseases and lesions which in your opinion would render butcher-meat unfit for human food.

*Therapeutics and Materia Medica.* Describe nitrate of potash. How is it obtained? Give its use, its action, its dose in the treatment of the horse and of the ox. Give the properties of salicylic acid. How is it obtained? Give its use, its action, its dose in the treatment of the horse, of the ox, and of the dog. Write a prescription for a cough mixture for the adult horse. What preparations of copper are used in veterinary medicine and for what purposes? What are the properties of plumbi acetas (sugar of lead)? How is it obtained? Give its action, its use, its dose in the treatment of the horse. Write a prescription for an astringent ointment containing a vegetable and a mineral ingredient. What is *oil of turpentine?* How is it obtained? Give its medicinal use and dose in the treatment of the horse and of the ox. What are *cantharides?* Where are they principally obtained and what are their active principles? Name *two* coal-tar products used to reduce temperature. What is the ordinary relative strength of a tincture to a fluidextract of the same drug? What are the medicinal uses of gentian root? Give the dose for the horse and for the ox. Define *laxative, saline purgative, drastic purgative, cholagogue purgative.* Give an example of each. Name *two* remedies that are commonly used to promote intestinal peristalsis. What is *Fowler's solution?* How is it prepared? Give dose for the ox, for the horse, and for the dog. Write a prescription for spasmodic colic.

The bill in the New York Legislature destined to relieve, through the Board of Regents, during a period of two months subsequent to the passage of the law, those graduates who neglected to register before the expiration of the time allowed by the act now in force in the Empire State, is reported on third reading in the lower branch as we go to press.

